# Philosophical Basis and Some Historical Aspects of Systems Biology: From Hegel to Noble - Applications for Bioenergetic Research

**DOI:** 10.3390/ijms10031161

**Published:** 2009-03-13

**Authors:** Valdur Saks, Claire Monge, Rita Guzun

**Affiliations:** 1 INSERM U884, Laboratoire de Bioénergétique Fondamentale et Appliquée, Université Joseph Fourier 2280 Rue de la Piscine, BP 53, Grenoble Cedex 9, France; E-Mails: Claire.Monge@bvra.ujf-grenoble.fr (C.M.); RGuzun@chu-grenoble.fr (R.G.); 2 Laboratory of Bioenergetics, National Institute of Chemical Physics and Biophysics, Akadeemia tee 23, 12618 Tallinn, Estonia

**Keywords:** Systems Biology, Molecular Systems Bioenergetics, cellular energy metabolism, regulation, compartmentation, energy transfer systems

## Abstract

We live in times of paradigmatic changes for the biological sciences. Reductionism, that for the last six decades has been the philosophical basis of biochemistry and molecular biology, is being displaced by Systems Biology, which favors the study of integrated systems. Historically, Systems Biology - defined as the higher level analysis of complex biological systems - was pioneered by Claude Bernard in physiology, Norbert Wiener with the development of cybernetics, and Erwin Schrödinger in his thermodynamic approach to the living. Systems Biology applies methods inspired by cybernetics, network analysis, and non-equilibrium dynamics of open systems. These developments follow very precisely the dialectical principles of development from thesis to antithesis to synthesis discovered by Hegel. Systems Biology opens new perspectives for studies of the integrated processes of energy metabolism in different cells. These integrated systems acquire new, system-level properties due to interaction of cellular components, such as metabolic compartmentation, channeling and functional coupling mechanisms, which are central for regulation of the energy fluxes. State of the art of these studies in the new area of Molecular System Bioenergetics is analyzed.

## Contents

1. Systems Biology – new paradigm and new perspectives of biological research.
2. Systems Biology and Hegel’s dialectic, some important steps in history.
3. Application of the Systems Biology approach to metabolic studies. Metabolic compartmentation as system level property.
4. Molecular System Bioenergetics: structural and dynamic organization of cellular energy metabolism: mitochondrial-cytoskeletal interactions, mitochondrial dynamics, energetic modules and regulation mechanisms.
5. Mathematical models of energy metabolism, useful and not very useful.

Wherever there is movement, wherever there is life, Wherever anything is carried into effect in the actual word, there Dialectic is at work. It is also the soul of all knowledge which is truly scientific.

Hegel’s Logic, Translated by William Wallace, Oxford University Press, Oxford UK, 2005, p. 116

## 1. Systems Biology – New Paradigm and New Perspectives of Biological Research

Within last decade, biological sciences have witnessed a radical change of paradigms [1–19]. Reductionism, which used to be a philosophical basis of biochemistry and molecular biology when everything – from genes to proteins and organelles – were studied in their isolated state is leaving its place to Systems Biology, which favours the study of integrated systems at all levels: molecular, cellular, organ, organism, and population [1–19]. The importance and rapid expansion of Systems Biology become clear when one opens PubMed with this keyword – tens of thousands of entries from different fields of the biological sciences appear. Hundreds, if not thousands of books have been published in recent years on this topic; references [1–9] are just very few examples of them, mostly related to the topics of this article. Indeed, suddenly Systems Biology is everywhere. Given the very rapidly increasing number of publications, it may even be that the term Systems Biology is not always understood in the same way, but nevertheless, there are general and rather precise commonly accepted definitions of this scientific direction. In 2005 Alberghina and Westerhoff edited a whole book analyzing the definitions and perspectives of Systems Biology [4]. The shortest and most clear definition of Systems Biology is given by Westerhoff’s group: “Systems Biology is the science that aims to understand how biological function absent from macromolecules in isolation, arises when they are components of their systems” [15]. Very similar definitions have been given by many other authors [1–19].

These very intensive developments make it interesting and necessary to discuss its origins – the philosophical basis and historical aspects of System Biology. This is especially interesting for scientists who have spent almost all of their time and efforts in studies of mechanisms of integrated cellular metabolism (not even knowing before that what they study is Systems Biology). For many of them the topics are very familiar, but what is most helpful for them is the development of new concepts within Systems Biology that help to make general conclusions and give new tools for further research. One of these areas of research concerns the study of integrated energy metabolism in cells which we call now Molecular System Bioenergetics, as one can see from the titles of this Special Issue and a recent volume published by Wiley VCH [7]. Metabolic studies are not new, for example studies of cardiac metabolism started more than 50 years ago, after pioneering works by Richard Bing [20]. The great value of Systems Biology for metabolic research is mostly conceptual because of the clear definition of system-level properties [1–10,15]. System-level properties are the results of interactions between components of the system [1,2,4,7,10–15]. Systems Biology gives also the tools for these studies, most important among them are the quantitative methods of modeling and network analysis [4–6,8–19] .

Already several books and articles have been written on the philosophy and origin of the systemic approach in biology [1–4,13,21,22]. Among them are recent publications by Noble [2,13,14], who has traced Systems Biology back to the works by Claude Bernard [23], concluding that Systems Biology is in fact physiology at new higher level [2,13,14] and that a genuine, quantitative theory of biology is to be developed in future research [14]. This is a very optimistic conclusion for new generation of scientists - they still have immense task ahead to work on, and this task attracts both attention and funding. Landmark precedents of Systems Biology are the work of N. Wiener associated with the development of cybernetics, along with the impact of E. Schrödinger’s contributions [24,25]. Another aim of this review is to show that the philosophical foundations of Systems Biology may be found in Hegel’s dialectical philosophy, as applied to biology [26–30]. Finally, a critical analysis of the current state of the art in Molecular System Bioenergetics will be given, in addition to those discussed in our recent book [7]. We have found this general presentation of Systems Biology and Molecular System Bioenergetics very useful for explaining and teaching these new disciplines to doctoral students at Grenoble University.

## 2. Hegel’s Dialectic and Systems Biology. Some Important Steps in History

According to Thomas Kuhn’s definition, a paradigm represents a specific, widely accepted way of viewing reality in science [26]. In this sense, Systems Biology is a new paradigm of biological sciences; it has been became widely popular within the last 10 – 15 years [1–19]. However, its history can be traced back into the two last centuries of biological and medical research, starting with Claude Bernard’s theory of permanence of the internal milieu of organisms, later called homeostasis [13,23]. From that time and up to the modern times of Systems Biology, life sciences appear to perfectly fit and strictly follow the dialectic principles of general historical developments discovered by Hegel [27–30].

### 2.1. Hegel’s dialectic laws

Georg Wilhelm Friedrich Hegel (1770 – 1831), one of most famous German philosophers, gave in his philosophy the most profound description of the logic and rules of historical developments [27–30]. An excellent description of this rather complicated philosophy is given by Bertrand Russell in his famous book “History of Western Philosophy” [30], explaining in easily way the dialectic laws. Hegel, as a most serious philosopher, was thinking about the relations of our thinking and the real world. For him, the real knowledge was to understand not only parts but the Whole, the Absolute Idea [27–30]. From our practical point of view (not to be involved in deep discussions between materialism and idealism in philosophy), Absolute Idea may be taken to represent the perfect, detailed knowledge of the integrated, whole systems. That in fact is what Systems Biology wants to find out, to know all about the life in its complexity, to comprehend the Whole, an Absolute Idea of the living systems. The process of development, the way to achieve this, the logic of finding out the Absolute Idea, according to Hegel, is the triadic movement called dialectic [30]. Dialectic consists of thesis, antithesis and synthesis [30]. Thus, knowledge as a whole has its triadic movement, and the process is essential to understand the results. To move from one stage to another, thinking as a dialectic process must “fall into the negative of itself” ([27], p. 35). Each later stage contains all the early stages, and all stages are given their proper place as a movement [30]. Thus, a thesis is first giving rise to its reaction, an antithesis which contradicts or negates the thesis, and the tension between the two being resolved by means of a synthesis [30].

**Figure 1.**
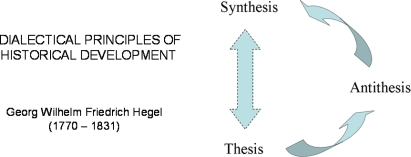
Hegelian dialectic of historical movement from thesis to antithesis to synthesis.

These dialectic movements were explained by Hegel and his followers by three basic concepts: 1) everything is made out of opposing forces/opposing sides (contradictions); 2) gradual changes lead to turning points, where one force overcomes the other (quantitative change leads to qualitative change); 3) change moves in spirals not circles (sometimes referred to as “negation of the negation”) [30]. These Hegelian dialectic rules are illustrated by the Scheme in Figure 1. Thomas Kuhn’s description of the structure of scientific revolutions [26] gives an excellent illustration of the second basic concept of Hegelian dialectic when applied to science. When we look into the history of Systems Biology, we find an excellent illustration of the validity of these dialectic laws. If Hegel had been a biologist, he could have predicted the appearance of Systems Biology as a necessary and inevitable step in our way to find out the final truth, the Absolute Idea of the life. What we know now is only the beginning of this long way.

### 2.2. Claude Bernard and the theory of permanence of internal milieu – homeostasis

Claude Bernard (1813–1878), a famous French physiologist, was a founder of experimental medicine, and according to Denis Noble [13] the first system biologist, one of the first instigators of Systems Biology (or integrative biology). The scope of his works was very wide: discovery of the pancreas function, discovery of the gluconeogenesis in liver, neurophysiology, toxicology, anesthesia and asphyxia. One of the main theories developed by Claude Bernard is the theory of the permanence of the *milieu intérieur* (later called homeostasis) due to integrated regulatory mechanisms. Analysis of the *milieu intérieur* is the study of the physiological mechanisms with which the organism can adapt itself to the *milieu extérieur* and maintain its functional balance in spite of the external constraints [23]. According to Claude Bernard “the fixity of the interior medium is the condition of a free and independent life” [23]. At his time, he had to separate himself and fight against popular theory of vitalism [21,22]. This theory postulated, in some way analogously with Systems Biology, that the whole living cell or organism is more than simple sum of its parts, but explained life by action of a vital force which neutralizes the “negative effects of physico-chemical forces” in living organism [21,22]. Bernard had to fight against this mystification of life [23]. He emphasized that an organism is able to adjust itself to external physical and chemical variations by maintaining permanence of its *milieu intérieur* and this adaptation is possible because the cells, the organs or the organisms are integrative systems. He was always attentive not to explain all his observations only by anatomy, claiming that anatomy has to serve physiology because of its complexity. He indicated that the function of an organ is not a strict one and that a function can be due to the interactions of two or several organs (for example the digestion process). According to him, physiologists must start from studies of physiological phenomena to explain them in the whole organism and not try to explain a function from an organ [23].

The strength of Claude Bernard’s theories comes also from his ability to extrapolate his works to chemistry, physics and mathematics. He understood the importance of the mathematical modeling to understand the natural phenomena because *“Cette application des mathématiques aux phénomènes naturels est le but de toute science, parce que l’expression de la loi des phénomènes doit toujours être mathématique” -* “*This mathematical application at natural phenomena is the aim of all sciences, because the expression of the laws of phenomena should always be mathematical”* [23]. And time proved that he was right. Applied mathematics, cybernetics and computer sciences are now very powerful tools in biological research. But Bernard understood also that the application of mathematical modelling should be based on very firm experimental data, which were not available at his time: *« C’est par elle seule [l’application mathématique] que, dans la suite, la science se constituera; seulement j’ai la conviction que l’équation générale est impossible pour le moment, l’étude qualitative des phénomènes devant nécessairement précéder leur étude quantitative. » « It is by it alone [the mathematical application] that, in the continuation, science will be created but I have the conviction that the general equation is impossible at the moment, the qualitative study of phenomena must necessary precede their quantitative study”* [23].

Thus, he understood the possible danger of misuse of the powerful method of mathematical modelling in biology: construction of mathematical models of a metabolic pathway or network can indeed lead to an erroneous model if the amount of physiological data is not sufficient. The richness of experimental data ensures the fidelity of the model. And we always have to keep in mind that a model is only a reflection of a complex system, a model will never be a system but only a representation of a system or a part of a system. The computer sciences are a very useful tool in Systems Biology but this tool must be used with caution inseparably from collection of experimental data, to avoid creating a virtual world far from reality. This very clever advice of Claude Bernard is still often forgotten or simply ignored in our times by a new generation of applied mathematicians coming into biological research with easy access to computing technologies but with rather weak knowledge of experimental data (see below). What we need to do first is to collect the maximally possible amount of experimental data describing the system level properties, with the aim to finally reach the “general equation” evoked by Claude Bernard. What did lack in Claude Bernard’s time was a sufficient amount of quantitative experimental data. Systems Biology is now in much more favourable position and following the advices given by Claude Bernard, we can go ahead taking up the challenge of finding the general equation of life, its Absolute Idea.

### 2.3. Cybernetics of Norbert Wiener and Systems Biology

Norbert Wiener (1894 – 1964) was an American mathematician who studied the communication and control processes both in technical electronic systems and in biology, notably in physiology, by analysis of information transmission and treatment processes [24]. He was a founder of cybernetics, a science of control and governing, which studies the structure and function of regulatory systems [24] and has very wide application in computer sciences, engineering, logic modeling as in electronic and information network (including Internet) theories, in physiology, evolutionary biology, neuroscience, anthropology, psychology, sociology. Application of cybernetics in biology is now known in general as biocybernetics, which is a part of theoretical biology, and plays a major role in systems biology, seeking to integrate different levels of information to understand how biological systems function. One of the most important achievements of cybernetics developed by Wiener was the theory of feedback regulation and its application for explanation of the mechanisms of homeostasis [24] discovered by Claude Bernard and described above. Discovery of the feedback mechanisms by Wiener is still probably the most important contribution of cybernetics into Systems Biology. Another direct application of biocybernetics is network biology [18,19]. As it was emphasized by Barabasi in his reviews on network biology, “quantifiable tools of network theory offer unforeseen possibilities to understand the cell’s internal organization and evolution, fundamentally altering our view of cell biology. The emerging results are forcing the realization that, notwithstanding the importance of individual molecules, cellular function is a contextual attribute of strict and quantifiable patterns of interactions between the myriad of cellular constituents” [19]. That tells us that understanding cell biology means understanding of system level properties.

### 2.4. Systems Biology: from Hegel to Noble

Systems Biology uses the methods of both experimental studies and computing, focusing on the studies of interactions within the system with the aim of understanding the biological function. In this vast area, there are many new particular directions of research, such as the Physiome Project [17]. And there are very numerous scientists and groups who have made significant contributions into this area [1–19]. The philosophy, general principles of these important studies on cellular and organ level have been analyzed and described by Denis Noble, who has summarized them in 10 basic principles, “10 commandments” [14].

**Figure 2.**
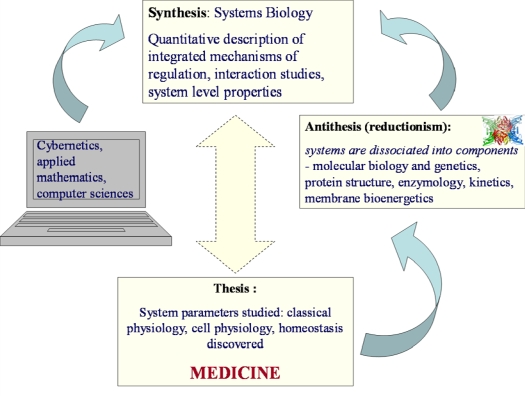
Presentation of development of biological sciences as Hegelian dialectic movement. In times of Claude Bernard, the problems of experimental physiology and medicine were formulated from the point of view of the theory of homeostasis at the organ level. To solve these problems, the components of the cell (proteins, genes, mitochondria etc.) were studied in the isolated state. In Systems Biology, these components are again studied in their interaction within the intact systems of interest.

Among these principles, transmission of information by feedback mechanisms is most important, but these mechanisms are to be discovered yet [2,14]. Thus, the whole process of development of biological sciences during last 150 years, from the times of Claude Bernard (experimental physiology, medicine) to that of molecular and structural biology, enzymology, membrane bioenergetics and then to Systems Biology perfectly follows the Hegelian dialectic principles, with triadic movement from thesis (formulation of problems of experimental biology and medicine) to antithesis (the systems are divided into components which are studied separately) to synthesis (coming back at new level to studies of biological function of whole system), as it is shown in Figure 2.

### 2.5. Erwin Schrödinger: negentropy production as a basis of metabolism, central role of bioenergetics

In the history of biological sciences, one of the most influential events has been publication of Erwin Schrödinger’s book “What is life?” in 1944 [25]. And it is still one of the most influential books in biology. Probably, it is not too much to say that scientists working in biology have successfully accomplished, with brilliant results, realizing the ideas described in the first chapters of this book related to foundation of molecular genetics, and are now busy in collectively reading the chapter 6 in this short book, related to the principles of organization of cellular metabolic processes, and all that together is now called Systems Biology.

**Figure 3.**
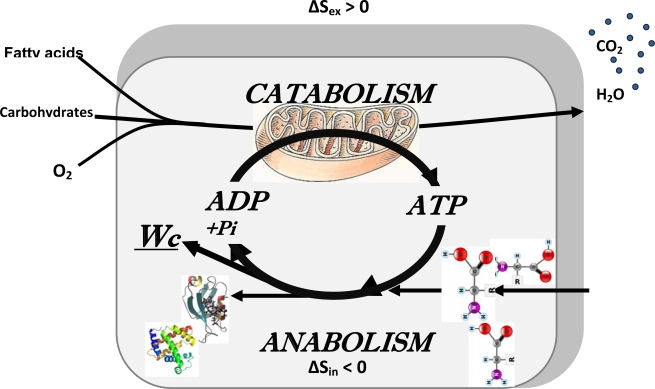
General scheme of cellular metabolism. A cell is a thermodynamically open system, in accordance with the Schrödinger’s principle of negentropy extraction. Increasing the entropy in extracellular medium, and decreasing it in the cell via metabolism is necessary for maintenance of the structural organization of both biopolymers as proteins, DNA and RNA, and also maintenance of the fine structural organization of the cell for effectively running compartmentalised metabolic processes. In this way, the cell can live in agreement with the thermodynamic laws (see the text). The Scheme shows the central role of bioenergetic processes in the cellular life by coupling catabolism with anabolism. Adapted from ref. [7].

The main conclusion made by Schrödinger was that the living cells need to be open systems with energy and mass exchange with surrounding medium, with the aim of maintaining their high structural and functional organization and thus internal entropy low, achieving this by means of increasing the entropy of the medium by catabolic reactions. Thus, Schrödinger wrote: “ The essential thing in metabolism is that the organism succeeds in freeing itself from all entropy it cannot help producing while alive” [25]. In cellular metabolic systems, catabolic reactions which increase entropy in surrounding medium are coupled to anabolic reactions (biosynthesis) which maintain cell structure and organization with necessary decrease in entropy. Catabolic reactions are mostly oxidative degradation of fatty acids and carbohydrates such as glucose. They are also the source of metabolic energy for the performance of any kind of cellular work. This is shown by the general scheme in Figure 3 describing the integrated metabolism of the cell as an open system exchanging both energy and masses with surrounding medium. This Scheme shows that the processes of free energy conversion are central for coupling catabolism to anabolism, emphasizing the central role of bioenergetics in studies of integrated metabolism of the cells. To live, the metabolic systems need to be in the steady state far from equilibrium, and how they maintain intracellular organization and low entropy state is explained by non-equilibrium thermodynamics: the organized states are maintained by energy and matter dissipation, therefore they are also known as *dissipative structures* [31–34].

## 3. Application of the Systems Biology Approach to Metabolic Studies. Metabolic Compartmentation as System Level Property

In studies of integrated metabolic processes, one of the most important problems is that of diffusion in the organized intracellular medium. In fact, all mathematical models of metabolism, and practical values of these models depend upon the authors’ views on cell structure and diffusion of metabolites, and still popular oversimplified theories of cell interior as a homogenous diluted solution of metabolites are sources of grave errors and may lead to meaningless models (see below). In our recent article in *International Journal of Molecular Sciences* we have analyzed in details the problems of diffusion of metabolites in organized intracellular medium [35]. Here, we emphasize some of the important conclusions made. The problem starts with the intracellular mobility of water, which is significantly reduced, leading to partitioning of metabolites between different water phases and to changes in binding constants [36–38]; then there is low-affinity adsorbtion of metabolites, especially if charged as ATP, to intracellular surfaces increasing the viscosity, and due to this the diffusion coefficient of metabolites is decreased by a factor of (1+C/K_d_)^−1^ where C is concentration of binding sites and Kd is dissociation constant of solute from these complexes [36]; macromolecular crowding and cytoskeletal structures create the barriers which increase the effective path-length of diffusion, and again diffusion coefficient is decreased by λ^−2^ where λ is relative increase in path attributable to the barriers [36–38]; finally, the movements of individual molecules become co-ordinated and vectorially directed due to organization of enzymes into the complexes, and the randomness of molecular events may be lost [36]. The results of these local diffusion restrictions are microcompartmentation of metabolites and their channeling within organized multienzyme complexes which need to be accounted for to explain biological phenomena [39,35]. Compartmentation and microcompartmentation of metabolites are system-level properties resulting from interactions between cellular components. Indeed, none of important observations in cellular bioenergetics could be explained by a paradigm describing a viable cell as a “mixed bag of enzymes” with homogenous metabolite distribution still sometimes in use: this simplistic theory excludes any possibility of metabolic regulation of cellular functions [35]. Due to macromolecular crowding and hindered diffusion cells need to compartmentalize metabolic pathways in order to overcome diffusive barriers. Biochemical reactions can successfully proceed and even be facilitated by metabolic channeling of intermediates due to structural organization of enzyme systems into organized multienzyme complexes. Metabolite channeling directly transfers the intermediate from one enzyme to an adjacent enzyme without the need of free aqueous-phase diffusion [40,41,7,42–46]. Enzymes are able to associate physically in non-dissociable, static multienzyme complexes, which are not random associations but an assembly of sequentially related enzymes, very often due to their association with cytoskeleton [42,43, 47]. Thus, principal mechanisms of functioning and regulation of cell metabolism are system-level properties: macro- and microcompartmentation, metabolic channeling and functional coupling, resulting from specific structural interactions between cellular components. For this reason Systems Biology approaches are most important for further advancement of metabolic studies. At the cellular level, it is becoming clear that most of biological characteristics arise from complex interactions between the cell’s numerous constituents, and based on protein-protein interactions, cellular metabolism is likely to be carried out in a highly modular manner within hierarchically organized networks [7,48]. The real problems and challenges for further studies are both to measure local concentrations of metabolites, including those of ATP in different cellular microcompartments and its metabolic channelling within microdomains (local fluxes), and to fully understand the nature of these restrictions of diffusion upon which intracellular compartmentation is based. This difficult work is necessary for reasonable computer modeling of the hierarchical modules of metabolic networks as a part of Molecular System Bioenergetics [7] and Systems Biology in general [11,17].

## 4. Molecular System Bioenergetics: Structural and Dynamic Organization of Cellular Energy Metabolism, Mitochondrial-Cytoskeletal Interactions, Mitochondrial Dynamics, Energetic Modules and Regulatory Mechanisms

Molecular Systems Bioenergetics is a science describing a new area of cellular bioenergetics in transition from molecular to the system level [7]. Miguel Aon has proposed the following definitions for this new direction of research [7]: *Molecular System Bioenergetics* is a broad research field accounting not only for metabolism as reaction networks but also for its spatial (organization) and temporal (dynamics) aspects. The main focus of Molecular System Bioenergetics are the processes of energy conversion both at molecular and cellular levels, with special emphasis on the structure and function of energy transfer and regulatory networks, mechanisms of interaction between their components, and a quantitative description of these networks by computational models. An important consequence of the organization of the enzymes into multienzyme complexes is vectorial metabolism and ligand conduction, a general principle proposed by Peter Mitchell after extensive enzymological studies and detailed characterization of mitochondrial proteins (reviewed in refs. [7] and [39]). The molecular system approach to the study of energy conversion in cells allows to fully explaining many classical observations in the cellular physiology of respiration, such as the metabolic aspects of the Frank- Starling law of the heart and the regulation of substrate supply to the cell [7, 49]. This approach helps us to understand how a cell senses its energy status in adjusting its functional activity under stressful conditions, or others aspects of its life.

### 4.1. Unitary organization of energy metabolism and compartmentalized energy transfer in cardiac cells

As we have seen above, coupling of catabolism with anabolism (the metabolism) is the way through which “negentropy” or free energy is extracted from the medium. Evolution has selected the adenine nucleotides to fulfil this important task of coupling catabolism and anabolism (Figure 3). A possible explanation is the rather high standard free energy change (ΔG° = 31.5 kJ/mol) and high affinity of ATP and ADP for many enzymes and carriers [50]. Cellular energetics is thus based on the reactions of ATP synthesis and utilization, *ADP* + *P_i_* ⇔ *ATP + H_2_O.* Taking water content as a constant and in excess (not changing in the reaction), the mass action ratio of the reaction of ATP synthesis is usually written as:

(1) $$\Gamma =\frac{\left[ATP\right]}{\left[ADP][P_{i}\right]}$$

By maintaining high mass action ratio for the reaction of ATP synthesis, catabolic reactions supply also free energy for cellular work. Free energy available in the cellular system is a function of the ratio of Γ to the equilibrium constant of the ATP synthesis, or:

(2) $$\Delta G_{ATP}=\Delta G_{ATP}^{0}+RT\;\text{ln}\;\frac{\left[ATP\right]}{\left[ADP][P_{i}\right]}$$

This function is usually called phosphorylation potential [51,52]. The principal purpose of free energy transformation associated to catabolic reactions is to keep a high value of the phosphorylation potential which is mostly achieved through mitochondrial oxidative phosphorylation or photosynthesis in autotrophic organisms. The theory of phosphorylation potential for the analysis of the cellular life was first used by Veech *et al*. [51] and Kammermeier *et al*. [52].

However, now it has become clear that applying Eq. 2 as well as any quantitative theory of physical chemistry to the real intracellular medium is not a simple task, namely due to the complex organisation of cell structure and metabolism. It has become clear that it is not the global ATP content which is important, but the ATP and the free energy available in micro- and macrocompartments which have to be accounted for, as it will be described below.

Studies by using pulsed-gradient ^31^P-NMR showed that the diffusion of ATP and phosphocreatine is anisotropic in muscle cells [53,54]. Recent mathematical modelling of the decreased affinity of mitochondria for exogenous ADP *in situ* in permeabilized cardiac cells also showed that the ADP or ATP diffusion in cells is heterogeneous and the apparent diffusion coefficient for ADP (and ATP) may be locally decreased (diffusion locally restricted) by an order, or even several orders of magnitude [55]. A similar limited diffusion of ATP in the subsarcolemmal area in cardiac cells was proposed by the Terzic and Dzeja group [56,57].

Application to cells of Eq. 2 in its general form is complicated by the compartmentation of ATP and adenine nucleotides, making the use of the easily measurable total ATP content very questionable and practically useless. ATP compartmentation has been studied in normal and ischemic heart for a long time (reviewed in ref. [7]), and recently demonstrated in several cell types by imaging techniques [58]. Most important recent data showing the significance of compartmentation phenomenon for cardiac energy metabolism have been collected by Neubauer’s group [59]. By using ^31^P-NMR spectroscopy in combination with imaging for investigation of cardiac muscle energy metabolism in patients, the authors showed that in the patients with cardiac disease – dilated cardiomyopathy (DCM) the decreased PCr/ATP ratio (lower than 1.6) is very clear and strong diagnostic index of increased mortality. In the heart of patients with DCM the ATP content remained the same as in healthy control patients, but PCr decreased by 70 % as compared to control. This shows the vital importance of the phosphocreatine – creatine kinase energy transfer network described below for the cardiac muscle normal function and life.

### 4.2. Cardiac cells as highly organized metabolic systems

Cardiac cells present a highly organized structure where mitochondria are localized at the A-band level within the sarcomere [60–62]. These cells represent best examples the complexity of organization of intracellular energy metabolism. Intermyofibrillar mitochondria are arranged in highly ordered crystal-like patterns in a muscle-specific manner, with relatively small deviations in the distances separating neighboring mitochondria [62,61]. Contrary to many other cells with less developed intracellular structures, dynamic changes in mitochondrial position due to their fission and fusion [63–65] are not found in adult and healthy cardiac and skeletal muscle cells because of their rigid intracellular structural organization, and mitochondria in these cells are morphologically heterogeneous (see the paper by Kuznetsov in this Special Issue). In this structurally organized medium, energy transfer between different subcellular micro- and macrocompartments (shortly called compartmentalized energy transfer) are of central importance. Existence of these rather complicated networks of energy transfer and signaling is a direct consequence of the compartmentalization of adenine nucleotides in the cells [55–58,66–69]. This is due to significant heterogeneity and local restrictions in the diffusion of adenine nucleotides in cells, and the necessity of rapid removal of ADP from the vicinity of MgATPases to avoid their inhibition by accumulating product – MgADP [7,70–77]. ATP is not only delivered by diffusion, but intracellular energy transfer is facilitated *via* networks consisting of phosphoryl-group transferring enzymes such as creatine kinase (CK), adenylate kinase (AK) and glycolytic phosphoryl-transferring enzymes [39,70–82]. Most important among them is the creatine kinase system. CK catalyzes the reversible reaction of adenine nucleotides transphosphorylation, the forward reaction of phosphocreatine (PCr) and MgADP synthesis and the reverse reaction of creatine (Cr) and MgATP production:

(3) $${\text{MgATP}}^{2-}+\text{Cr}↔{\text{MgADP}}^{-}+{\text{PCr}}^{2-}+{\text{H}}^{+}$$

Four CK isoforms, each with compartmentalized cellular location, exist in mammals. Specific mitochondrial CK isoenzymes (MtCK), called ubiquitous (uMtCK) and sarcomeric (sMtCK), are functionally coupled to oxidative phosphorylation and produce PCr from mitochondrial ATP. PCr in turn is used for local regeneration of ATP by the muscle cytoplasmic isoform of CK (M-CK), driving myosin-ATPases or ion pump-ATPases [ 39,70–82].

**Figure 4.**
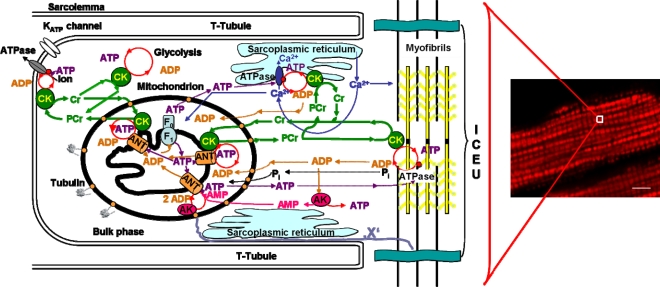
Organization of compartmentalized energy transfer and metabolism in cardiac cells by intracellular energetic units (ICEU). Adapted from ref. [7]. The scheme shows the structural organization of the energy transfer networks of coupled CK and AK reactions within an ICEU. By interaction with cytoskeletal elements, the mitochondria and sarcoplasmic reticulum (SR) are precisely fixed with respect to the structure of sarcomere of myofibrils between two Z-lines and correspondingly between two T-tubules. Calcium is released from SR into the space in ICEU in the vicinity of mitochondria and sarcomeres to activate contraction and mitochondrial dehydrogenases. Adenine nucleotides within ICEU do not equilibrate rapidly with adenine nucleotides in the bulk water phase. The mitochondria, SR and MgATPase of myofibrils and ATP sensitive systems in sarcolemma are interconnected by metabolic channeling of reaction intermediates and energy transfer within ICEU by the creatine kinase – phosphocreatine and myokinase systems. The protein factors (still unknown and marked as “X”), most probably connected to cytoskeleton, fix the position of mitochondria. One of these proteins – tubulin-also controls the permeabilty of the VDAC channels for ADP and ATP. Adenine nucleotides within ICEU and bulk water phase may be connected by some more rapidly diffusing metabolites as Cr – PCr. Mitochondria were labelled by pre-incubation of cells with mitochondrial inner membrane potential sensitive probe MitoTracker Red (50 nM). Very regular arrangement of mitochondria and thus ICEUS is seen. Scale bar 10 μm.

In the heart as well as in oxidative skeletal muscle the intracellular energy transfer networks are structurally organized in the intracellular medium where macromolecules and organelles, surrounding a regular mitochondrial lattice, are involved in multiple structural and functional interactions [81–83]. Figure 4 summarizes available information about such an organized and compartmentalized energy metabolism in cardiac cells. This scheme also illustrates the view that mitochondria in muscle cells are structurally organized into functional complexes with myofibrils and sarcoplasmic reticulum [81–83]. These complexes were called ‘intracellular energetic units”, ICEUs, and taken to represent the basic pattern of organization of muscle energy metabolism [81].

There are no physical barriers between ICEUs, each mitochondria (or several adjacent mitochondria) can be taken to be in the centre of its own ICEU. This concept is consistent with very regular, crystal like arrangement of mitochondria in cardiac cells [60–62] and describes the organized functional connections of mitochondria with their neighbours. ICEUs are analogous to calcium release units, (CRUs), structurally organized sites of Ca^2+^ microdomains (Ca^2+^ sparks) which form a discrete, stochastic system of intracellular calcium signaling in cardiac cells [84,85].

This concept is supported by Weiss *et al*. [48], who presented a holistic view of cardiovascular metabolism, considering it from the perspective of a physical network, in which various metabolic modules are spatially distributed throughout the interior of the cell to optimize ATP delivery to specific ATPases [48]. In addition to a mitochondrial module (which is represented by ICEUs) the authors considered also a module consisting of glycolytic enzyme complexes serving for energy channeling to molecular complexes in sarcolemma and sarcoplasmic reticulum, and modules of calcium cycling (which Wang *et al*. called calcium release units, CRU [84]). These modules were further analyzed from the abstract perspective of fundamental concepts in network theory and dynamic perspective of interactions between modules [48]. Understanding the nature of these interactions within hiercharchical modular structures is a main challenge of research of cardiac metabolism to gain deeper understanding of possible mechanisms of cardioprotection [48].

Metabolic compartmentation described above and unitary organization of energy metabolism is clear and important examples of system-level properties. Identification of cell components responsible for specific organization of energy transfer systems and intracellular diffusion merits further investigation. One of these components may be tubulin which is able to bind to the VDAC channels in mitochondrial outer membrane and in this way to decrease the apparent affinity of mitochondria for ADP [86, 87]. For further elucidation of the nature of cytoskeletal components responsible for specific organization of both mitochondrial arrangement and complex metabolic signalling and intracellular energy transfer pathways, further investigations on the proteome are needed.

**Figure 5.**
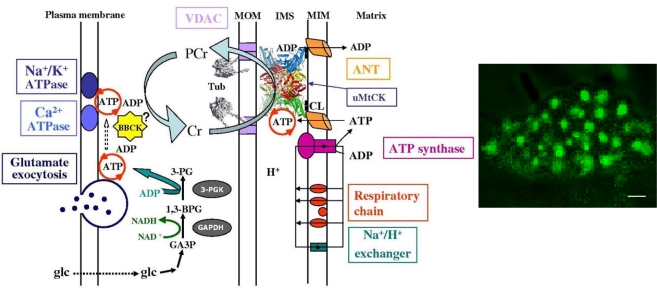
Energetics of brain synaptosomes. Sites of ATP production (mitochondrial matrix) and sites of ATP consumption (ion transport across the plasma membrane and vesicle trafficking for neurotransmitter uptake and release, e.g. glutamate) are linked by an energy transfer pathway represented by the phosphocreatine/creatine kinase system. uMtCK bound to mitochondrial inner membrane (MIM) via cardiolipin (black squares). ATP consumed by the energy consuming reactions is reproduced locally by BBCK from PCr. GA3P, glyceraldehyde-3-phosphate; 1,3-BPG, 1,3 biphosphoglycerate; 3-PG, 3-phosphoglycerate; GAPDH, glycerate-3-phosphate-deshydrogenase; 3-PGK, 3-phosphoglycerate kinase; VDAC, voltage dependant anion channel; MOM, mitochondrial outer membrane; uMtCK, ubiquitous mitochondrial creatine kinase; Tub – αβ heterodimer of tubulin interacting with the VDAC channels and limiting its permeability for adenine nucleotides. Right panel shows the confocal image of isolated rat synaptosomes Mitochondria were labelled by pre-incubation with mitochondrial inner membrane probe MitoTracker Green (50 nM). Scale bar 1 μm. Adapted from reference [88].

The energy transfer network similar to that described above for cardiac cells is also functioning in brain cells, particularly in synaptosomes (Figure 5) [88].

### 4.3. Unitary organization of energy metabolism versus mitochondrial reticulum

Another important question is related to the role and cell specificity of mitochondrial fusion and fission. Recent works in this area showed that the mitochondrial fusion and fission are potentially important for cell differentiation and pathophysiology [63, 89–92]. Many proteins responsible for fission and fusion such as Dynamin-related protein-1 (DRP1), Mitofusin-1 and Mitofusin-2 (Mnf1, Mnf2), OPA1 – have been identified [90–93], as being involved in cancer or apoptosis, during mitochondrial mobility changes by disrupting cytoskeletal architecture and in some other human pathologies.

All these numerous studies on mitochondrial dynamics, fusion and fission have been carried out mostly in yeast or various cultured cells (easy to grow and use in confocal microscopic studies), when the cells are usually at the stage of continuous division. Probably for this reason, the general conclusion has been made that fusion and fission phenomena are characteristic and necessary for normal functioning of mitochondria [90–92]. This general and rather enthusiastic conclusion is, however, not totally justified. Many works [61,94–99] have shown that fusion and fission of mitochondria are not observed in non-dividing adult cardiomyocytes. Thus, the fusion and fission are not necessary for normal functioning of mitochondria and for cardiac cell energetics in particular. Instead, mitochondrial localization and regular arrangement in muscle cells are controlled by cytoskeleton [86,87,100–102]. By its nature, the contraction process needs very precise structural organization of sarcomeres and muscle cells [7]. Changes in the lattice spacing between actin and myosin filaments in sarcomeres due to alteration of titin orientation are the basis of length-dependent activation of sarcomere contraction and Frank-Straling law [49]. Mitochondria in muscle cells are in fixed positions determined by their interactions with cytoskeleton and also with sarcoplasmic reticulum. Cytoskeleton plays an important role for mitochondrial and cell morphology and motility, intracellular traffic, mitosis [103,104]. Complex cytoskeletal network (microfilaments, microtubules, intermediate filaments) with specific cytoskeleton associated proteins interacts with mitochondria [86,87,97,100,105,106]. Very recently, Rostovtseva *et al*. have shown the ability of tubulin to bind directly to the VDAC channel on mitochondrial outer membrane and to control the permeability of this channel [86–88]. These interactions are thought to be responsible for mitochondrial regular arrangement into unitary structures (energetic modules, ICEUs) [48,81]. It is not yet completely clear; however, which components of the cytoskeleton and to which extent are responsible for the arrangement of mitochondria in cardiomyocytes into regular networks with modular organization. Organized and regularly arranged energetic units (modules) in adult cardiomyocytes represent a good example of regular networks, while NB HL-1 and many other cells [107] are examples of irregular networks.

It has to be mentioned that the rather popular hypothesis according to which the mitochondrial fusion is a necessary requirement for their normal function [90–92] evidently contradicts all the 50 years of experimental evidence in bioenergetics. During these five decades all laboratories all over the world have isolated mitochondria from heart, liver, skeletal muscle, brain etc. in perfectly granular shape and intact smooth outer and dynamic inner membranes [108–111] in functionally intact state, with all soluble Krebs cycle substrates including NADH in the matrix. If the mitochondria were in the cells in fused state as proposed by Twig *et al*. [92], rapid homogenisation of tissue should disrupt all mitochondrial membranes and release Krebs cycle substrates from matrix, and isolated mitochondria should represent only membrane fragments not capable to use Krebs-cycle linked substrates as pyruvte or glutamate malate – but this is never the case if isolation is performed carefully. Thus, systemic approach requires taking into account all available data before any general conclusion can be made; taking out only some fragmented data is nothing more than a relic of classical reductionism.

The very regular arrangement of distinct mitochondria into modular structures (ICEUs) evidently serves an important purpose of survival of heart cells under stress conditions. Under these conditions depolarization and functional damage of separate mitochondria does not result in complete breakdown of cell energetics, since contractile function of the heart is still maintained by other energetic units which continue to function in a well synchronized manner. The mechanism of this synchronization is still not precisely known and is under active studies in several laboratories [33,94,95,112]. On the contrary, fusion of mitochondria in heart cells is a clear sign of pathogenesis and cell death. Using electron microscopy Sun *et al*. showed already in 1969 that in perfused heart hypoxia resulted in formation of gigantic mitochondria due to the fusion process [113]. Thus, in cardiomyocytes mitochondrial fusion is most probably the beginning of their degradation and energetic breakdown of the cells.

### 4.4. Excitation-contraction coupling and cardiac energetics: membrane energy sensing

**Figure 6.**
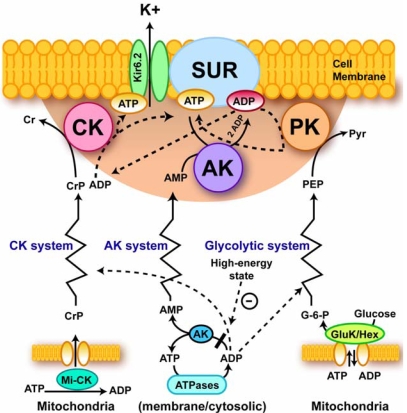
A paradigm of phosphotransfer-mediated energetic signaling: coupling cellular metabolic and electrical activities. Dynamic interaction between creatine kinase (CK), adenylate kinase (AK) and glycolytic (represented by pyruvate kinase, PK) phosphotransfer relays determines a prototypic metabolic sensor - K_ATP_ channel behavior and subsequent cellular responses, such as excitability, hormone secretion, intracellular calcium homeostasis and vascular tone. The shadowed area represents a metabolic sensor “sensing zone”, where intimate local changes in nucleotide ratios are sensed and transduced into an appropriate cellular response. Phosphotransfer circuits connect the “sensing zone” with cellular processes. Dashed lines indicate pathways signaling the high-energy state, while solid lines represent low-energy state signal transmission. Kir6.2 – potassium channel subunit; SUR – sulfonylurea receptor; GluK/Hex –glucokinase and hexokinase. Reproduced from ref. [7] with permission.

For modeling of the heart function within the projects of Systems Biology (as Physiome project), it is important to quantitatively describe relationships between energy metabolism and electrical activity of the cells. There is a clear need to account for the system level properties, such as metabolic compartmentation and channeling phenomena, in modeling the energy sensing of ion currents across sarcolemma [49, 80]. In the control of the excitation – contraction coupling in the heart a principal step is the sarcolemmal membrane metabolic sensor complex [56,57,114–116]. Its main component is the sarcolemmal ATP sensitive K^+^ (K_ATP_) channel acting as an alarm system to adjust cell electrical activity to the metabolic state of the cell [56,57,114–116]. The sarcolemmal MM CK creatine kinase rephosphorylates the local ADP maintaining a high ATP/ADP level in these microcompartments for coordination of membrane electrical activity with cellular metabolic status, notably with PCr levels (see Figure 6).

The K_ATP_ channel was discovered by Noma, and it was found that this channel has high affinity for ATP, about 100 μM [117,118]. Nevertheless, the channel is opened in the presence of millimolar ATP, as seen from rapid membrane repolarisation and shortening of action potential in ischemic and hypoxic hearts or as shown directly in experiments with internally perfused cardiomyocytes [56,57]. This is explained by strong diffusional restriction and thus ATP compartmentation in the subsarcolemmal area [119] and linked to the cellular pool of PCr via CK reactions. Pool exhaustion in the first minutes of ischemia certainly contributes to the cessation of contraction due to opening of K_ATP_ channel and decreased calcium entry. This energy transfer and control functions are shared by the whole system, including the creatine kinase, the adenylate kinase and glycolytic systems, as it was seen in experiments involving genetic manipulation [79,120,121]. Similar cell-membrane metabolic sensors may be also important in brain cells. The phosphotransfer relays communicate metabolic signals originating in mitochondria or at cellular ATPases to metabolic sensors conveying information about “high” or “low” cellular energy, oxygen supply or hormonal states [56,122].

Metabolic feedback signaling by phosphotransfer networks described above explains quantitatively the metabolic aspect of the classical Frank-Starling law regarding regulation of cardiac function and respiration under conditions of metabolic stability and unchanged calcium transients and changes of cardiac function in ischemia [49]. These systems are based on the compartmentalized energy transfer, whose deficit explains the rapid fall of contractile force in the first minutes of total ischemia. By regulating the sarcolemmal metabolic sensor - K_ATP_ channels, the CK-AK-glycolytic network affects the excitation-contraction process and the calcium cycle of the cell [80,39]. The latter system explains adrenergic modulation of cardiac cell function and energetics under stress [123–130]. Both systems may be activated simultaneously, as it is observed in the case of positive inotropy induced by β-adrenergic agents, when Frank-Starling curves are shifted upward [49]. The physiological mechanism of respiration regulation described above has the important advantage of ensuring effective control of free energy conversion across the whole physiological range of workloads, without requiring a severe increase in cytoplasmic calcium and ADP concentrations. It thus avoids any danger of mitochondrial calcium overload that would open the mitochondrial permeability transition pore and thus lead to cell death [49]. Functioning of the coupled MtCK – ANT system in mitochondria prevents from the reactive oxygen species (ROS, oxygen free radicals) formation in mitochondrial respiratory chain and helps to avoid many problems related to ROS production, such as PTP opening, necrosis, apoptosis and rapid ageing [131]. In this way, the CK – PCr network may significantly contribute in the positive effects of physical exercise on the human health: exercise – induced increased fluxes via this pathway increase the ADP-ATP turnover in the coupled MtCK-ANT reactions in mitochondria and keeps the ROS production low.

Thus, effective cardiac work and fine metabolic regulation of respiration and energy fluxes need the organized and interconnected energy transfer and metabolic signaling systems. Direct transfer of ATP and ADP between mitochondria and different cellular compartments is not able to fulfill this important task efficiently.

### 4.5. Molecular system analysis of integrated mechanisms of regulation of fatty acid and glucose oxidation

Molecular system analysis as a method is also useful for elucidation of the mechanisms of regulation of substrate supply for the heart [132]. In muscle cells, contractile function and cellular energetics are fuelled by oxidation of carbohydrate substrates and fatty acids [133–135]. The choice of substrates depends upon their availability, and the rates of their utilisation are very precisely regulated by multiple interactions between the intracellular compartmentalized and integrated bioenergetic systems of glycolysis, fatty acid oxidation and the Krebs cycle in the mitochondrial matrix, linked directly to the activity of the respiratory chain and the phosphorylation process catalysed by the ATP synthase complex [132,134]. The rates of all these processes are geared to the workload, mostly by the mechanism of the feedback metabolic regulation described above [132,134].

**Figure 7.**
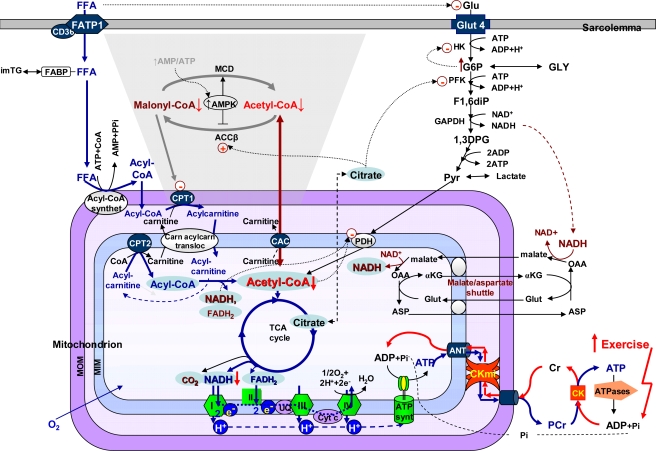
The scheme of substrate supply for mitochondrial respiration and the mechanisms of feedback regulation of the fatty acid and glucose oxidation during workload elevation in oxidative muscle cells: central role of TCA cycle intermediates. Reproduced from reference [132] with permission.

The network of reactions of main substrate supply for mitochondrial respiration in muscle cells and their multiple interactions and feedback mechanisms of regulation are illustrated in Figure 7. The choice of the substrates for oxidation depends on their availability, and if glucose and FFA are both present, FFA strongly inhibits the transport of glucose across the plasma membrane both in heart and in skeletal muscle [132,134,135]. At relatively low workloads, mitochondrial acetyl-CoA and NADH produced by beta-oxidation tend to inhibit the pyruvate dehydrogenase complex in the mitochondrial inner membrane, and citrate, the production of which is increased in the Krebs cycle, after transport across the inner mitochondrial membrane into the cytoplasm, inhibits PFK [134]. A glucose-fatty acid cycle (Randle hypothesis): if glucose and FFA are both present, FFA inhibit the transport of glucose across the plasma membrane, acyl-CoA oxidation increases the mitochondrial ratios of acetyl-CoA/CoA and of NADH/NAD^+^, which inhibit the pyruvate dehydrogenase (PDH) complex, and increased citrate (produced in the TCA cycle) can inhibit phosphofructokinase (PFK). These changes would slow down oxidation of glucose and pyruvate (PYR) and increase glucose-6-phosphate (G6P), which would inhibit hexokinase (HK), and decrease glucose transport. The mitochondrial creatine kinase (miCK) catalyzes the direct transphosphorylation of intramitochondrially produced ATP and cytosolic creatine (Cr) into ADP and phosphocreatine, (PCr). ADP enters the matrix space to stimulate oxidative phosphorylation, while PCr is transferred via cytosolic Cr/PCr shuttle to functional coupling of CK to ATPases (acto-myosin ATPase and ion pumps), resulting in release of high free energy of ATP hydrolysis. If the workload increases, ATP production and respiration are increased due to feedback signalling via creatine kinase (CK) system, leading to decrease of mitochondrial acetyl-CoA content, which is transferred into cytoplasm with participation of carnitine acetyl carrier (CAC). Acetyl-CoA carboxylase (ACC) is responsible for converting acetyl-CoA to Malonyl-CoA, a potent inhibitor of CPT-I, with the aim to avoid overloading the mitochondria with fatty acid oxidation intermediates, when the workload is decreased. Inactivation of ACC occurs via phosphorylation catalyzed by AMP-activated protein kinase (AMPK). Phosphorylation and inactivation of ACC leads to a decrease in the concentration of malonyl-CoA. A fall in malonyl-CoA levels disinhibits CPT1, resulting in increased fatty acid oxidation. Malonyl-CoA is also converted back into acetyl-CoA in the malonyl–CoA decarboxylase (MCD) reaction. Increase in the workload increases the rate of acetyl-CoA consumption and that automatically decreases the malonyl-CoA content. The ACC and MCD regulation occur under stress conditions when the AMP/ATP ratios are increased, but are unlikely to occur under normal work-load conditons of the heart. Thus, AMPK may be envisaged as a modulator, under situations of cellular stress, rather than as a master on-off switch of fatty acid oxidation.

It is the Krebs cycle, which is on the crossroads between the metabolic pathways of glucose and fatty acid oxidation, and its intermediates that play a very important role of feedback metabolic regulation of upstream pathways of substrate oxidation [132,134].

FFAs are taken up by a family of plasma membrane proteins (fatty acid transporter protein (FATP1), fatty acid translocase (CD36) and in cytoplasm associated with fatty acid binding protein (FABP). FFAs are esterified to acyl-CoA via fatty acyl-CoA synthetase. The resulting acyl-CoA is the transported through the inner membrane of the mitochondrion, via the exchange of CoA for carnitine by carnitine-palmitolyltransferase I (CPT I). Acylcarnitine is then transported by carnitine-acylcarnitine translocase into the mitochondrial matrix where a reversal exchange takes place through the action of carnitine-palmitoyltransferae II (CPT II). Once inside, the mitochondrion acyl-CoA is a substrate for the beta-oxidative pathway, resulting in acetyl-CoA production. Each round of beta-oxidation produces one mole of NADH, one mole of FADH_2_ and one mole of acetyl-CoA. Acetyl-CoA enters the TCA cycle, where it is further oxidized to CO_2_ with the concomitant generation of three moles of NADH, one mole of FADH_2_ and one mole of ATP. Acetyl-CoA which is formed in the mitochondrial matrix, can be transferred into the cytoplasm with participation of carnitine, carnitine acetyltransferases and carnitine acetyltranslocase (carnitine acetylcarnitine carrier complex, CAC).

Glucose (GLU) is taken up by glucose transporter-4 (GLUT-4) and enters the Embden-Meyerhof pathway, which converts glucose via a series of reactions into two molecules of pyruvate (PYR). As a result of these reactions, a small amount of ATP and NADH are produced. G6P – glucose 6-phosphate, HK – hexokinase, PFK – phosphofructokinase; GLY – glycogen; F1,6diP – fructose-1,6-bisphosphate, GAPDH – glyceralhehydephosphate dehydrogenase, 1,3DPG – 1,3-diphosphoglycerate. The redox potential of NADH is transferred into the mitochondrial matrix via the malate/aspartate shuttle; OAA – oxaloacetate, Glut – glutamate, αKG – alpha – ketoglutarate, ASP - aspartate. Malate generated in the cytosol enters the matrix in exchange for alpha-ketoglutarate (αKG) and can be used to produce matrix NADH. Matrix oxaloacetate (OAA) is returned to the cytosol by conversion to ASP and exchange with glutamate (Glut). Most of the metabolic energy derived from glucose can come from the entry of pyruvate into the citric acid cycle and oxidative phosphorylation via the acetyl-CoA production. NADH and FADH_2_ are oxidized in the respiratory chain (complexes I, II, III and IV). These pathways occur under aerobic conditions. Under anaerobic conditions, pyruvate can be converted to lactate.

## 5. Mathematical Models of Energy Metabolism, Useful and Not Very Useful

Because of the high complexity of the processes involved, mathematical modeling is an increasingly important part of Systems Biology, including Molecular System Bioenergetics. Different models of cardiac energy metabolism have already been used to analyze the mechanisms of regulation of respiration and cellular energy fluxes [136–145], with rather contradictory results. There is no complete model available as yet, no “general equation” (as Claude Bernard could call it) of energy metabolism, and this will need further intensive work. However, the number of models developed is already significant, whch allows us to try to classify them by their impact and constructive contribution into our understanding of regulation of integrated metabolism, and to critically analyze some errors which have become evident. In general, the models can be easily classified as good, bad and very bad. Bad and very bad models are those which authors have ignored the wise advice of Claude Bernard to take into account the maximally possible amount of reliable experimental data (see above), and thus have only some virtual value. An extreme case of these models is that by Barros and Martinez [146] who consider the cell as a sphere where a metabolite is produced by a single source and diffuses freely in a homogenous, isotropic medium, the cytosol. The authors take the diffusion coefficients for metabolites determined for bulk water phase as the only realistic values, which for ATP would be equal to about 500 μm^2^/s [146]. The fact that these values for diffusion coefficients may be significantly different inside cells with a protein concentration of approximately 100–200 mg/mL were not considered, thus the possibility that physical barriers resulting from a dense cytoskeletal or mitochondrial network or from macromolecular crowding [35] were not taken into account at all. Taking this for granted, the authors conclude that “even under most favorable conditions that are compatible with the known physical constraints, it would be impossible that ATP pools could appear in the cytosol of a compact cell”, and then conclude that “unrealistic conditions were needed to form ATP domains” [146]. No experimental data were analyzed to verify the correctness of this model and the conclusions derived. Critical analysis of this model can be found in our previous article in IJMS [35]. The problem is that Barros and Martinez are not alone – there is whole group of authors who have produced models of cardiac energy metabolism based on the theory of homogenous cytoplasm as diluted solution of metabolites. Some of them (critically analysed in [147]) arrive at non-realistic conclusion that the cells must be much bigger than they are (these are very bad, non-realistic models). Some other authors [148,149] by applying otherwise correct theories of chemical thermodynamics of dilute solutions have recently discovered that the cardiac function is governed by a general, average value of phosphorylation potential (see equation 2). These authors ignore the pioneering experimental works of Kammermeyer’s and Radda’s groups [52,150]. In the beginning of metabolic research, including studies by use of ^31^P-NMR method, the first problem to study was to investigate the question how changes of cardiac function might be dependent on average phosphorylation potential, and the result was very clear: there is no dependence, cardiac function can be changed manifold at almost constant value of phosphorylation potential [52,150]. Thus, the modeling result [149] contradicts experimental data – there is no way to call this model a good one. The reason is evidently that the model does not account for the compartmentation phenomenon described above.

The models which describe the mitochondrial metabolism, including respiratory chain and Krebs cycle, are in much better situation and have given some of the important results, consistent with experimental data – evaluation of the role of calcium in regulation of mitochondrial activities, role of Pi in respiration regulation [138–140]. Most important is to apply these models correctly for in vivo conditions taking into account the compartmentation phenomenon.

Finally, there are the compartmentalized energy transfer models aimed at describing quantitatively the results of *in vivo* studies of respiration regulation in the ICEUs under physiological conditions of the Frank-Starling law. These models are based on the concepts of ICEUs and include: kinetics of ATP hydrolysis by actomyosin ATPase during contraction cycle, diffusional exchange of metabolites between myofibril and mitochondrial compartments, VDAC-restricted diffusion of ATP and ADP across mitochondrial outer membrane, the mitochondrial synthesis of ATP by ATP synthase, ΔpH and ΔΨ controlled Pi and ADP transport into mitochondrial matrix, PCr production in the coupled mitochondrial CK reaction and its utilization in cytoplasmic CK reaction [136,137]. These events are considered in a system consisted of a myofibril with a radius of 1 um, a mitochondria, and a thin layer of cytoplasm interposed between them [136,137]. The modeling results show cyclic changes in the concentration of ADP in the core of myofibrils in ICEUs in a microcompartment containing myofibrillar-bound MM-CK, where ADP is first produced by actomyosin MgATPase during the contraction cycle of crossbridges, and then rephosphorylated by the CK due to a non-equilibrium state of the CK reaction [136,137]. Interestingly, these calculated cyclic changes in PCr, ATP and Cr which are in the range of 5–10 % of their cellular contents, are in good agreement with the multiple observations of the cyclic changes of these compounds in the contraction cycle published in the literature [151,152]. These changes in Cr, PCr and total ATP are however close to the experimental errors of their detection, thus giving an overall impression of metabolic stability. Changes in ADP and Pi concentrations are relatively much more significant because of very low initial values. Without CK, the changes of local ADP concentrations in these microcompartments will be much more dramatic [136,137]. Within the whole contraction cycle the rates of ADP and ATP cycling and thus the respiration in mitochondria coupled to the PCr production are increased with elevation of the workload. Increasing cyclic changes in the local ADP production in myofibrils are immediately displacing the myofibrillar MM-CK reaction in the direction of local ATP regeneration. The amplitude of displacement of CK from equilibrium is proportionally increased with workload [136,137]. In this regard, CK, adenylate kinase and other phosphotransfer isoenzymes in different intracellular compartments are “pushed” or “pulled” from the equilibrium in opposite directions, depending on the activity of an associated process which drives steady-state high-energy phosphoryl flux [49]. The model quantitatively describes the experimental observations on the dependence of the respiration rate upon the workload [7,49]. Evidently, to find the “general equation”, the complete model of integrated energy metabolism of muscle and brain cells we need to develop these constructive models which have already given us significant explanation of experimental data. The more complete model(s) should include all data and phenomena shown in Figures 4 – 7. Only these models can be included into more general project of Systems Biology and Molecular System Bioenergetics.

## 6. Conclusions

Systems Biology as a new paradigm of biological sciences which favours the study of integrated systems at all levels: cellular, organ, organism, and population with the aim of explaining biological function by interaction of system components provides new conceptual tools for studies of integrated metabolic processes. The aim of Systems Biology is the higher-level analysis of complex biological systems by using the wealth of information obtained in studies of isolated components, applying the methodological approaches of cybernetics, applied mathematics, network analysis, nonequilibrium thermodynamics of open systems. From a historical perspective the first systems biology approach was already applied by Claude Bernard about 150 years ago, and further important contributions were made by Norbert Wiener and Erwin Schrödinger. The developments of biological research during last 150 years follow very precisely the dialectical principles of development from thesis to antithesis to synthesis discovered by Hegel. The Systems Biology opens new perspectives for studies of the integrated processes of energy metabolism in different cells. These integrated systems acquire new, system-level properties due to interaction of cellular components, such as metabolic compartmentation, channeling and functional coupling mechanisms, which are central for regulation of the energy fluxes. All these mechanisms are functioning within phosphotransfer networks of the compartmentalized energy transport. These mechanisms explain such important physiological phenomena as metabolic aspects of Frank-Starling law of the heart and membrane sensing of cellular energy levels. Mathematical modeling of these important systems is a promising approach in Molecular System Bioenergetics.
